# RNA-seq of muscle from pigs divergent in feed efficiency and product quality identifies differences in immune response, growth, and macronutrient and connective tissue metabolism

**DOI:** 10.1186/s12864-018-5175-y

**Published:** 2018-11-01

**Authors:** Justyna Horodyska, Klaus Wimmers, Henry Reyer, Nares Trakooljul, Anne Maria Mullen, Peadar G. Lawlor, Ruth M. Hamill

**Affiliations:** 1 Teagasc, Food Research Centre, Ashtown, Dublin, 15 Ireland; 2 Leibniz Institute for Farm Animal Biology (FBN), Institute for Genome Biology, Dummerstorf, Germany; 3 Faculty of Agricultural and Environmental Sciences, University Rostock, Rostock, Germany; 4 Teagasc, Pig Development Department, AGRIC, Moorepark, Fermoy, Co. Cork, Ireland

**Keywords:** FE, RFI, Residual feed intake, Gene expression, Transcriptomics, RNA

## Abstract

**Background:**

Feed efficiency (FE) is an indicator of efficiency in converting energy and nutrients from feed into a tissue that is of major environmental and economic significance. The molecular mechanisms contributing to differences in FE are not fully elucidated, therefore the objective of this study was to profile the porcine *Longissimus thoracis et lumborum* (LTL) muscle transcriptome, examine the product quality from pigs divergent in FE and investigate the functional networks underpinning the potential relationship between product quality and FE.

**Results:**

RNA-Seq (*n* = 16) and product quality (*n* = 40) analysis were carried out in the LTL of pigs differing in FE status. A total of 272 annotated genes were differentially expressed with a *P* < 0.01. Functional annotation revealed a number of biological events related to immune response, growth, carbohydrate & lipid metabolism and connective tissue indicating that these might be the key mechanisms governing differences in FE. Five most significant bio-functions altered in FE groups were ‘haematological system development & function’, ‘lymphoid tissue structure & development’, ‘tissue morphology’, ‘cellular movement’ and ‘immune cell trafficking’. Top significant canonical pathways represented among the differentially expressed genes included ‘IL-8 signalling’, ‘leukocyte extravasation signalling, ‘sphingosine-1-phosphate signalling’, ‘PKCθ signalling in T lymphocytes’ and ‘fMLP signalling in neutrophils’. A minor impairment in the quality of meat, in relation to texture and water-holding capacity, produced by high-FE pigs was observed. High-FE pigs also had reduced intramuscular fat content and improved nutritional profile in terms of fatty acid composition.

**Conclusions:**

Ontology analysis revealed enhanced activity of adaptive immunity and phagocytes in high-FE pigs suggesting more efficient conserving of resources, which can be utilised for other important biological processes. Shifts in carbohydrate conversion into glucose in FE-divergent muscle may underpin the divergent evolution of pH profile in meat from the FE-groups. Moreover, altered amino acid metabolism and increased mobilisation & flux of calcium may influence growth in FE-divergent muscle. Furthermore, decreased degradation of fibroblasts in FE-divergent muscle could impact on collagen turnover and alter tenderness of meat, whilst enhanced lipid degradation in high-FE pigs may potentially underlie a more efficient fat metabolism in these animals.

**Electronic supplementary material:**

The online version of this article (10.1186/s12864-018-5175-y) contains supplementary material, which is available to authorized users.

## Background

Pork consumption accounts for over 36 percent of the world’s meat intake [1]. Porcine muscle is a significant source of high biological value proteins, vitamins and minerals, as well as dietary fats such as saturated fatty acids (SFA), monounsaturated fatty acids (MUFA), polyunsaturated fatty acids (PUFA), cholesterol and triacylglycerol [2]. SFA and cholesterol content, have been linked to obesity, cardiovascular disease and type 2 diabetes mellitus [3, 4], therefore consumers perceive leaner pork, which is lower in these components as a more healthy option [5, 6].

Feed efficiency (FE) is an indicator of efficiency in converting energy and nutrients from feed into a tissue that is of major nutritional and economic significance [7]. FE is a complex trait involving many organs and can be influenced by environmental and health related factors [8, 9]. Skeletal muscle, being the largest organ in the body and an important location of carbohydrate and lipid metabolism [10–12], plays a particularly important role in the utilisation and storage of a large proportion of the energy acquired from feed. Therefore enhancing our understanding of the biological processes occurring in muscle from FE-divergent pigs could optimise the strategies to improving FE and ease the production cost and ecological footprint from pork production. Furthermore, FE has been shown to be associated with product quality and nutritive profile in several studies, with evidence that the muscle of high-FE pigs exhibits reduced adiposity [13, 14], lower SFA and MUFA, and an enhanced proportion of PUFA [14], which is known for its protective properties against cardiovascular disease [15], and altered overall product quality [13, 14, 16, 17]. Thus divergence in FE is not only of importance to animal production, but it also impacts consumers’ preference with regards to quality, nutritive value and wholesomeness of meat.

The molecular mechanisms contributing to differences in FE are not fully elucidated. To date, few studies have conducted transcriptome profiling of skeletal muscle in FE-divergent pigs e.g. [18–20]. Furthermore, these studies did not examine the consequences of divergence in FE on product quality. Here we investigate the impact of divergence in residual feed intake (RFI; the difference between actual feed intake and predicted feed requirements) on product quality of the porcine *Longissimus thoracis et lumborum* (LTL) muscle. Furthermore, we identify in that muscle important biological functions and pathways enriched with differentially expressed (DE) genes in relation to FE, and the functional networks underpinning the relationship between product quality and FE.

## Results

### Differential gene expression profile

**Fig. 1:**
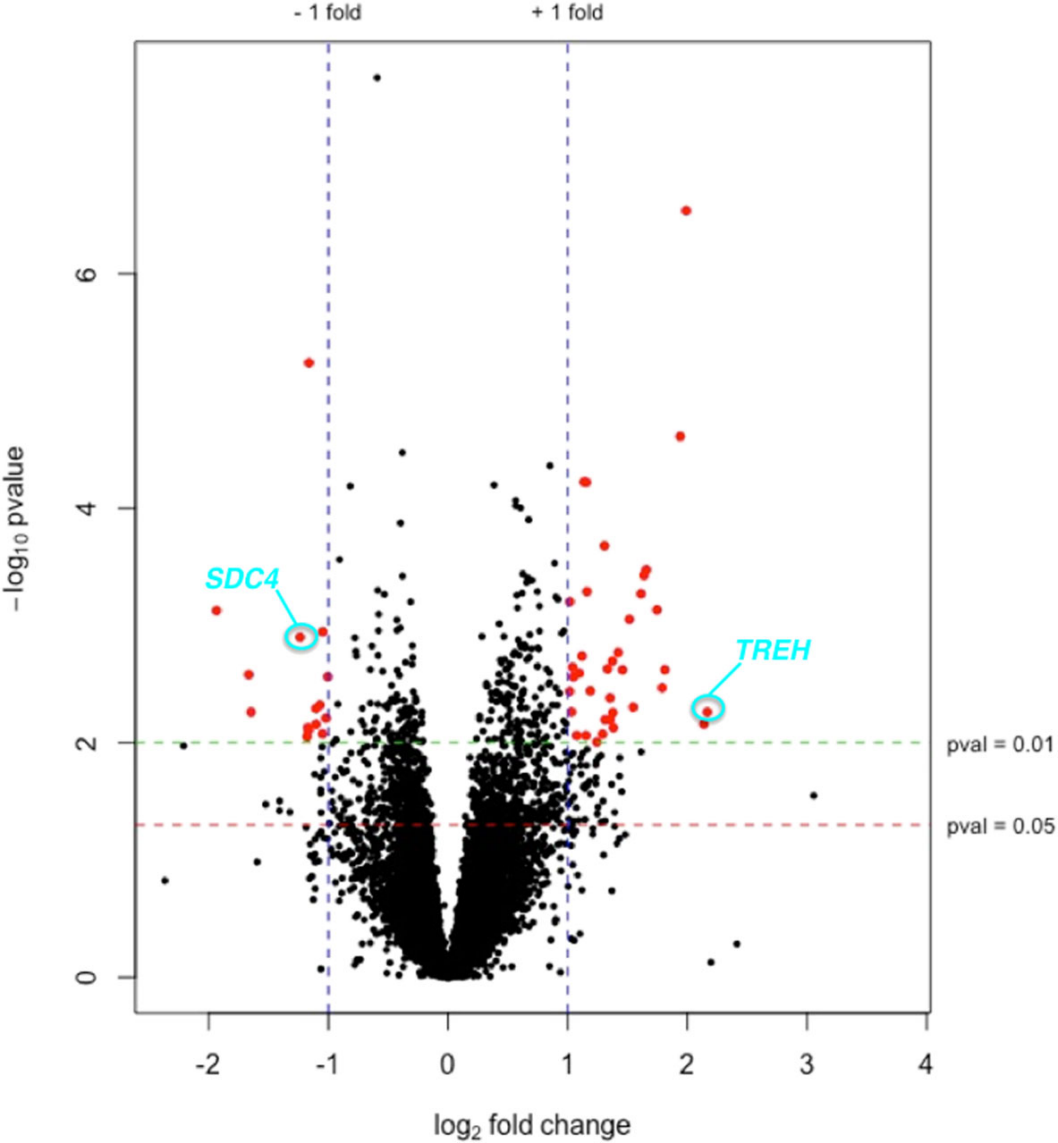
Volcano plot depicting a total of 14,497 genes expressed in muscle from pigs divergent in feed efficiency. The horizontal green and red lines indicate the suggestive significance thresholds of differentially expressed (DE) genes at *P* < 0.01 and 0.05, respectively. The vertical blue lines represent the threshold of log2 fold change ≥ |1| (fold change ≥ |2|) and the red dots depict significantly DE genes at *P* < 0.01 and log2 fold change ≥ |1| (fold change ≥ |2|). Positive and negative fold changes refer to up- and down-regulated genes in high-FE pigs, respectively. The most up- and down-regulated annotated genes are highlighted in a circle

An average of 104.4 million high quality paired-end reads per sample were mapped to the reference with a mean of 80.9% mapping efficiency. A total of 14,497 genes were expressed in the muscle (Fig. 1) and of these 306 (272 annotated) genes were differentially expressed between high- and low-FE samples with a *P* < 0.01 corresponding to false discovery rate (q) ≤ 0.47. Of these annotated genes, 176 were up- and 96 were down-regulated, whilst 140 were found to be at least 1.5-fold differentially expressed in high- versus low-FE pigs (Additional file 1: Table S1). The most altered genes were *TREH* (fold change = 4.49; high-FE > low-FE) and *SDC4* (fold change = -2.35; high-FE < low-FE). Transcripts with a *P* < 0.01 corresponding to a q ≤ 0.47 were considered significantly differentially expressed, which is not a highly stringent cut-off because the differences in mRNA abundances between the FE groups were relatively small. However to offset this lower statistical stringency for differential expression profiling, B-H corrected p-values were used to refine the data that was further utilised to extract bio-functions, pathways and networks.

### Gene ontology analysis

**Fig. 2:**
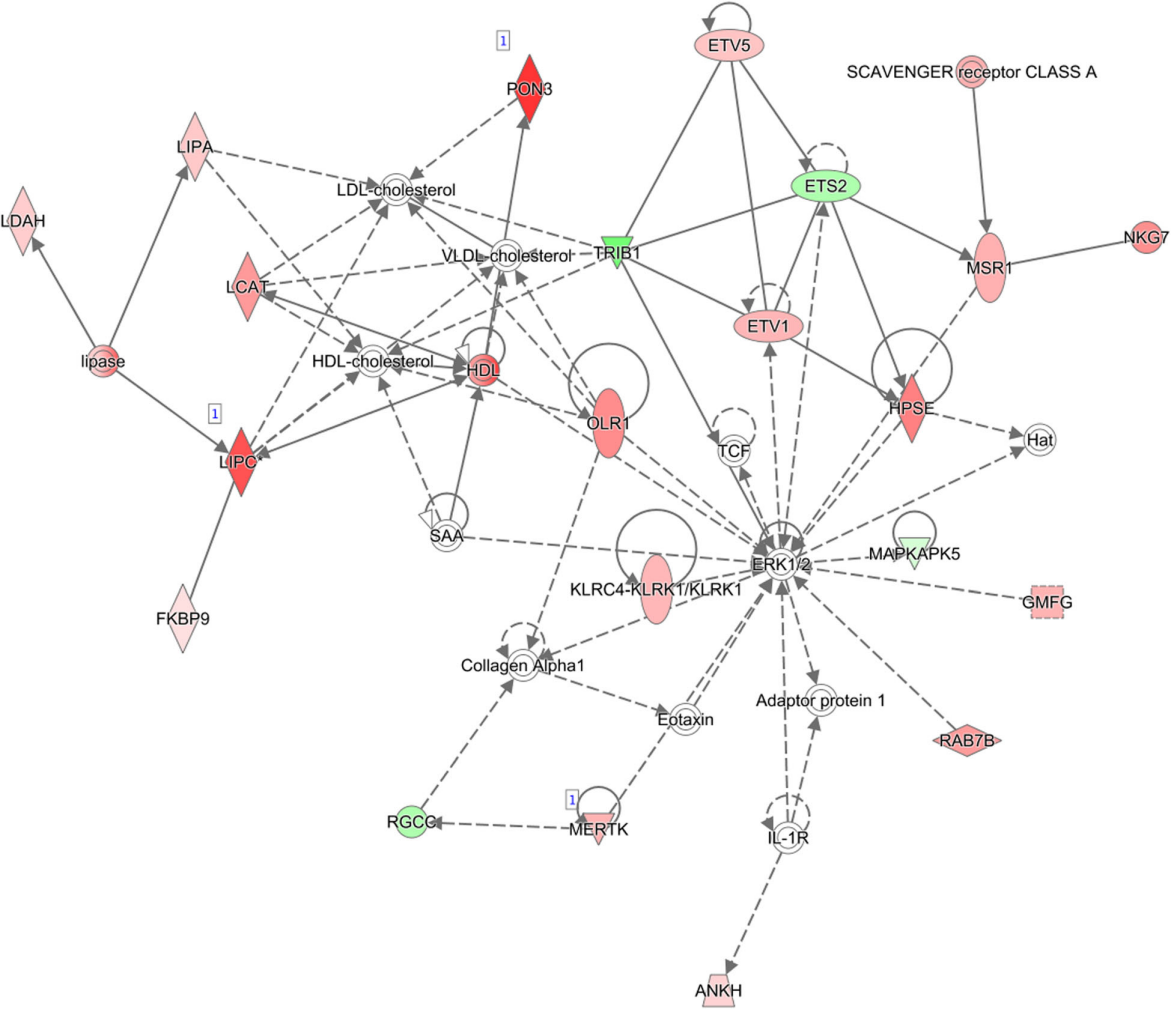
Network #2 containing functions related to ‘protein synthesis’, ‘lipid metabolism’, and ‘molecular transport’. Genes are denoted as nodes and the biological relationship between two nodes is denoted as an edge/line. Node colour represents up- (red) and down- (green) regulated genes in high-FE pigs

Enrichment analysis of the DE genes was utilised to investigate biological processes and pathways altered in response to differences in FE. Thirty nine biological functions and thirty eight canonical pathways were significantly (*P* < 0.01) enriched with DE genes. Most affected biological functions were ‘haematological system development & function’, ‘lymphoid tissue structure & development’, ‘tissue morphology’, ‘cellular movement’ and ‘immune cell trafficking’ (Table 1). A list of sub-categories enclosed within each function is presented in Additional file 2: Table S2. A number of functions ranged from significantly repressed to significantly activated state, including ‘haematological system development and function’ (z-score range: -2.13 – 3.52), ‘tissue morphology’ (z-score range: -2.13 – 2.87) and ‘cell death and survival’ (z-score range: -2.27 – 2.20). Functions containing a positive z-score range included ‘immune cell trafficking’ (z-score range: 0.58 – 3.52), cell-to-cell signalling and interaction’ (z-score range: 0.12 – 3.19), cell-mediated immune response’ (z-score range: 0.58 – 2.94), ‘tissue development’ (z-score range: 0.78 – 2.30) and ‘vitamin and mineral metabolism’ (z-score range: 2.36 – 2.89). Furthermore, most affected pathways were ‘IL-8 signalling’, ‘leukocyte extravasation signalling’, ‘sphingosine-1-phosphate signalling’, ‘PKCθ signalling in T lymphocytes’ and ‘fMLP signalling in neutrophils’ (Table 2 and Additional file 3: Table S3). Analysis of molecule connectivity revealed nineteen networks enriched with DE genes, of which network #2 (Fig. 2) contained 21 DE molecules related to macronutrients metabolism, specifically ‘protein synthesis’, ‘lipid metabolism’ and ‘molecular transport’.

**Table 1:** Molecular, cellular and physiological categories significantly over-represented among the differentially expressed genes

Category	B-H *p*-value range*	Z-score range^a^
Haematological System Development & Function	1.72E-11 – 5.00E-03	-2.13 – 3.52^b^
Lymphoid Tissue Structure & Development	1.72E-11 – 5.00E-03	-0.65 – 2.94^b^
Tissue Morphology	1.72E-11 – 5.16E-03	-2.13 – 2.87^b^
Cellular Movement	1.72E-11 – 5.74E-03	-0.36 – 3.52^b^
Immune Cell Trafficking	6.54E-11 – 5.74E-03	0.58 – 3.52^b^
Cellular Function & Maintenance	6.54E-11 – 4.56E-03	-0.06 – 2.83^b^
Cellular Development	1.29E-10 – 5.00E-03	-0.65 – 1.64
Cellular Growth & Proliferation	1.29E-10 – 5.00E-03	-0.65 – 1.73
Cell-To-Cell Signalling & Interaction	2.21E-09 – 5.74E-03	0.12 – 3.19^b^
Protein Synthesis	6.50E-09 – 2.32E-03	0.24 – 1.18
Humoral Immune Response	9.96E-09 – 3.58E-03	-0.57 – 1.89
Cellular Compromise	1.14E-08 – 2.80E-03	-1.51 – 2.20^b^
Cell-mediated Immune Response	1.79E-07 – 5.74E-03	0.58 – 2.94^b^
Free Radical Scavenging	2.33E-07 – 8.89E-07	1.13
Cell Death & Survival	2.42E-06 – 4.57E-03	-2.27 – 2.20^b^
Tissue Development	3.60E-06 – 4.56E-03	0.78 – 2.30^b^
Embryonic Development	5.77E-06 – 3.53E-03	0.54 – 1.50
Haematopoiesis	5.77E-06 – 1.11E-03	0.78 – 1.77
Organ Development	5.77E-06 – 3.94E-03	-0.66 – 1.50
Organismal Development	5.77E-06 – 4.95E-03	0.72 – 1.64
Cell Morphology	1.39E-05 – 4.95E-03	-0.08 – 2.09^b^
Lipid Metabolism	1.48E-05 – 1.19E-03	-1.23 – 0.60
Small Molecule Biochemistry	1.48E-05 – 5.74E-03	-1.23 – 0.60
Organ Morphology	1.70E-05 – 4.95E-03	1.06 – 1.77
Molecular Transport	1.97E-05 – 1.81E-03	-0.01 – 2.89^b^
Cardiovascular System Development & Function	2.63E-05 – 4.95E-03	-0.95 – 2.12^b^
Digestive System Development & Function	6.90E-05 – 5.38E-03	-0.66
Organismal Survival	8.09E-05 – 8.09E-05	-0.04
Cell Signalling	1.05E-04 – 1.81E-03	0.12 – 3.19^b^
Vitamin & Mineral Metabolism	1.05E-04 – 1.81E-03	2.36 – 2.89^b^
Cell Cycle	5.91E-04 – 3.94E-03	-0.49 – -1.98
Gene Expression	5.91E-04 – 3.53E-03	-1.98 – -0.49
Cellular Assembly & Organization	7.30E-04 – 5.74E-03	0.33 – 1.89
Renal & Urological System Development & Function	1.74E-03 – 3.94E-03	-0.15 – 0.76
Carbohydrate Metabolism	2.09E-03 – 2.09E-03	NA
Amino Acid Metabolism	2.74E-03 – 5.74E-03	NA
Hepatic System Development & Function	3.20E-03 – 3.20E-03	-0.66
Skeletal & Muscular System Development & Function	3.54E-03 – 3.54E-03	NA
Nervous System Development & Function	4.63E-0 – 4.63E-03	NA

*Range of B-H multiple testing correction *p*-values of enriched functions within the category; ^a^range of z-scores for sub-categories contained within a particular category; ^b^annotations with a z-score > 2 and z-score < -2 were considered significantly activated and inhibited in high-FE pigs, respectively; NA: no activity pattern available

**Table 2:** Most significant canonical pathways observed among differentially expressed genes in relation to feed efficiency (FE)

Canonical Pathway	-log (B-H p-value)	Z-score	Genes
IL-8 signalling	4.13	2.50^a^	***RND2*** *,* ***PIK3C2B*** *,* ***PLCB2*** *,* ***VCAM1*** *,* ***PTK2B*** *,* ***PIK3C2G*** *,* ***GNB5*** *, MAPK8,* ***RAC3*** *, ROCK2,* ***ITGB2*** *,* ***FGFR4*** *,* ***NCF2*** *,* ***CYBB***
Leukocyte extravasation signalling	3.38	1.73	***PIK3C2B*** *,* ***VCAM1*** *,* ***PTK2B*** *,* ***CXCR4*** *,* ***PIK3C2G*** *, MAPK8, RAPGEF3, ROCK2,* ***ITGB2*** *,* ***FGFR4*** *,* ***NCF2*** *,* ***CYBB*** *,* ***VAV1***
Sphingosine-1-phosphate signalling	3.38	1.67	***RND2*** *,* ***PIK3C2B*** *,* ***NAAA*** *,* ***PLCB2*** *,* ***PTK2B*** *,* ***FGFR4*** *, ADCY4,* ***PIK3C2G*** *, SPHK1,* ***CASP1***
PKCθ signalling in T lymphocytes	3.22	0.63	***PIK3C2B*** *, MAP3K14,* ***FGFR4*** *,* ***PIK3C2G*** *, MAPK8,* ***CD86*** *, NFATC2,* ***VAV1*** *,* ***RAC3*** *, NFATC1*
fMLP signalling in neutrophils	2.86	1.41	***PIK3C2B*** *,* ***PLCB2*** *,* ***FGFR4*** *,* ***NCF2*** *,* ***PIK3C2G*** *,* ***CYBB*** *,* ***GNB5*** *, NFATC2, NFATC1*
B cell receptor signalling	2.86	0.91	***PIK3C2B*** *, MAP3K14, FOXO1,* ***PTK2B*** *,* ***FGFR4*** *,* ***PIK3C2G*** *, MAPK8, NFATC2,* ***VAV1*** *,* ***PIK3AP1*** *, NFATC1*
Myc mediated apoptosis signalling	2.86	NA	*MYC,* ***PIK3C2B*** *,* ***FGFR4*** *,* ***PIK3C2G*** *, MAPK8, CYCS,* ***SFN***
Chemokine signalling	2.86	1.13	*ROCK2,* ***PLCB2*** *,* ***PTK2B*** *,* ***CXCR4*** *,* ***PIK3C2G*** *, MAPK8,* ***CCL5***
Gαq signalling	2.86	1.00	***RND2*** *, ROCK2,* ***PIK3C2B*** *,* ***PLCB2*** *,* ***PTK2B*** *,* ***FGFR4*** *,* ***PIK3C2G*** *,* ***GNB5*** *, NFATC2, NFATC1*
CD28 signalling in T helper cells	2.86	0.33	***PIK3C2B*** *,* ***FGFR4*** *,* ***PIK3C2G*** *, MAPK8,* ***CD86*** *, NFATC2,* ***VAV1*** *,* ***CTLA4*** *, NFATC1*

^a^Significantly activated (z-score > 2) pathways in high-FE pigs, *NA* no activity pattern available, up-regulated genes in high-FE pigs are highlighted in bold and down-regulated genes in normal typeface

### Validation of RNA-seq results

Expression patterns of thirteen genes (*FAM134B*, *FOXO1*, *SPP1, TRIM63*, *CASP1*, *COL11A1*, *CYBB*, *ITGB2*, *MYC*, *PLCB2*, *SDC4*, *TREH* and *VCAM1*), which were selected randomly and also as representatives of the canonical pathways altered by feed efficiency, were confirmed through qPCR. *RPL10* and *RPL32* were utilised as reference genes to normalise expressions of these target transcripts. Significant differences in the mRNA abundances of *FAM134B*, *FOXO1*, *TRIM63*, *COL11A1*, *CYBB*, *MYC*, *PLCB2*, *SDC4*, *TREH* and *VCAM1* transcripts between the FE groups were verified. Moreover, *CASP1* and *ITGB2* exhibited a *P* < 0.1. Spearman correlations between RNA-seq and qPCR data, ranging from 0.509 to 0.950, were significant for all thirteen assessed mRNAs (Table 3).

**Table 3:** Comparison of RNA-seq and qPCR data of selected genes affected by feed efficiency (FE)

Gene	qPCR fold change	RNA-seq fold change	Spearman’s rho
*FAM134B*	2.1**	2.1**	0.874***
*FOXO1*	1.6***	1.5**	0.785***
*SPP1*	**3.6**	2.5*	0.950***
*TRIM63*	2.2**	2.0**	0.918***
*CASP1*	**1.8** ^£^	1.9**	0.509*
*COL11A1*	1.8*	1.5**	0.968***
*CYBB*	1.5*	1.6**	0.641**
*ITGB2*	**1.7** ^£^	1.5*	0.765**
*MYC*	-1.5*	-1.6**	0.947***
*PLCB2*	1.5*	1.7**	0.612*
*SDC4*	-2.5***	-2.4**	0.868***
*TREH*	3.5*	4.5**	0.818***
*VCAM1*	1.4*	1.4**	0.803***

^£^*P* < 0.1, *P < 0.05, **P < 0.01, ***P < 0.001; up-regulated genes in high-FE pigs are highlighted in bold and down-regulated genes in normal typeface

### Product quality

**Fig. 3:**
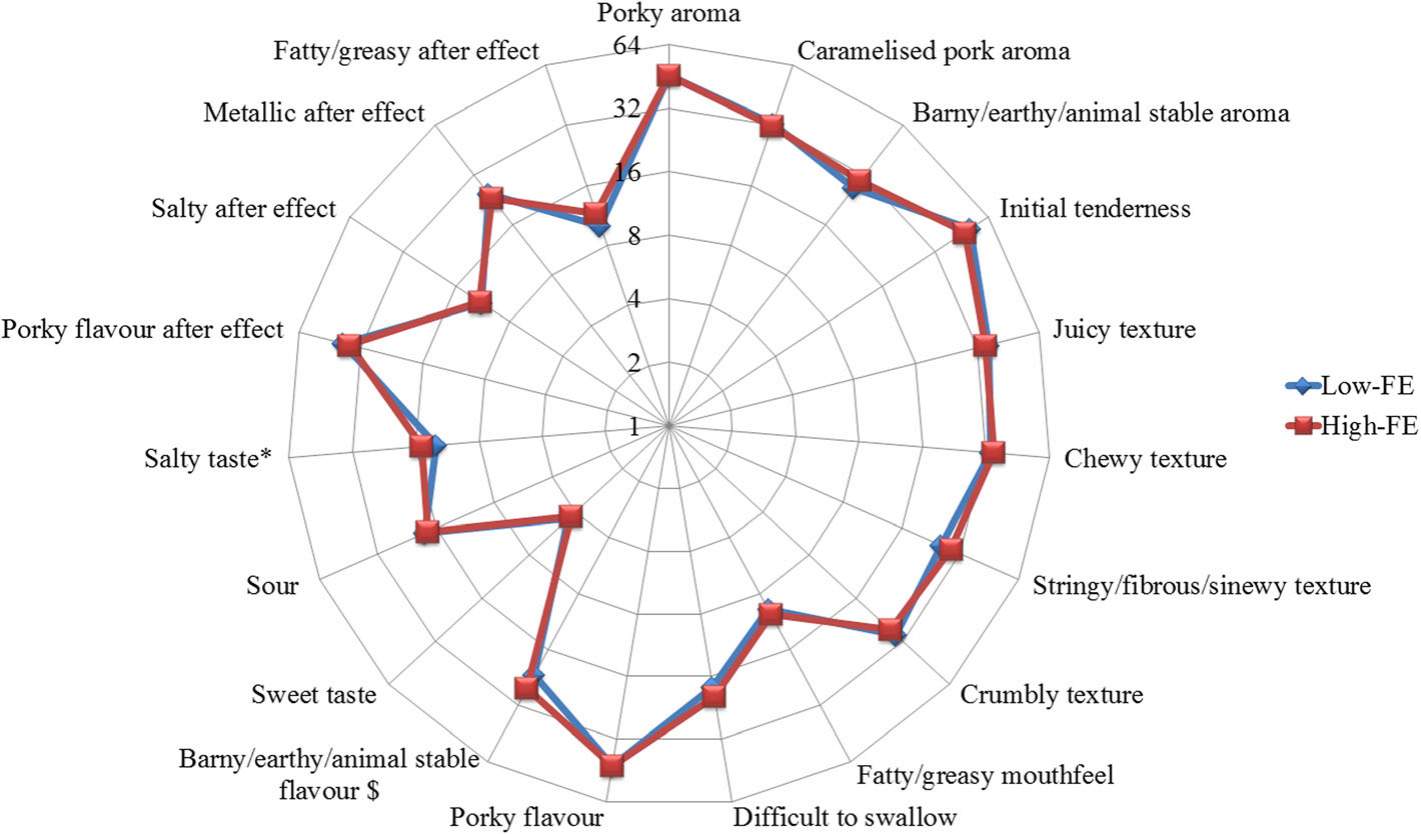
Radar chart illustrating meat sensory attributes of *Longissimus thoracis et lumborum* muscle from FE-divergent pigs. Panellists scored meat from 0 (not detectable) to 100 (extremely detectable). ^$^*P* < 0.1, *P < 0.05

**Fig. 4:**
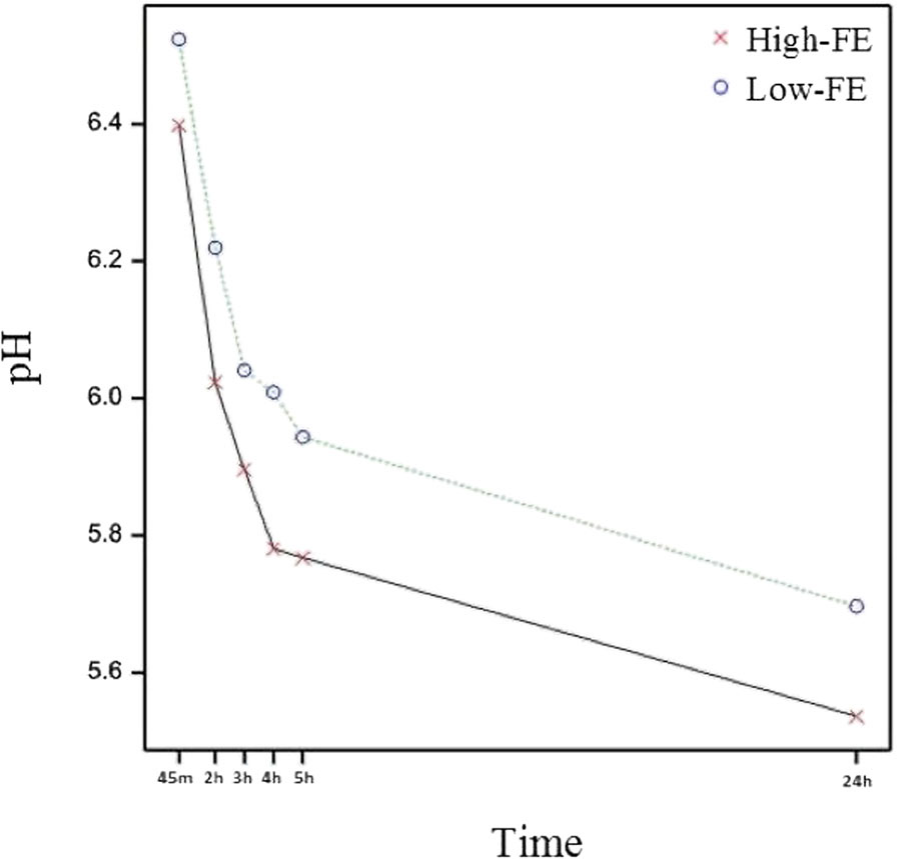
Timeplot depicting *post-mortem* pH evolution of *Longissimus thoracis et lumborum* muscle divergent in feed efficiency. pH 45m: *P* < 0.1; pH 2h, 3h, 4h, 5h, 24h: *P* < 0. 05.

**Fig. 5:**
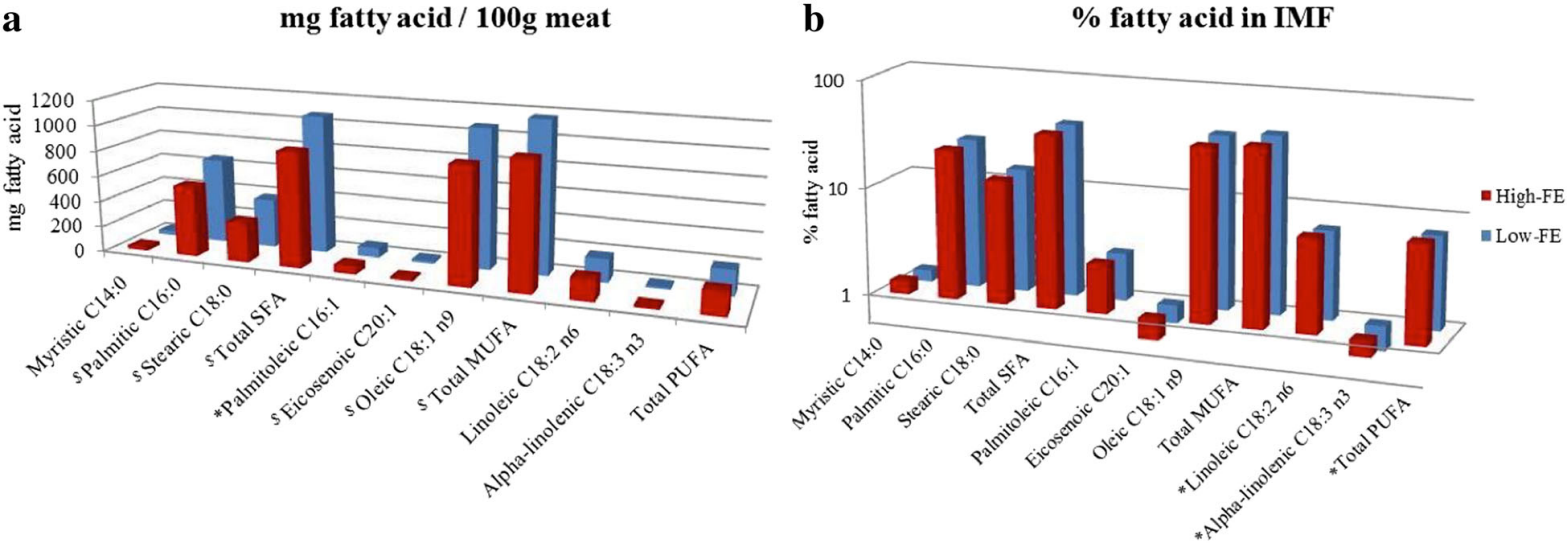
Bar chart illustration of fatty acids composition of pigs divergent in feed efficiency (FE). Bar chart (**a**) displays fatty acid composition in *Longissimus thoracis et lumborum* muscle and (**b**) shows percentage of fatty acid in intramuscular fat (IMF). SFA: saturated fatty acids, MUFA: monounsaturated fatty acids, PUFA: polyunsaturated fatty acids. ^$^*P* < 0.1, *P < 0.05

Carcass and product quality traits of FE-divergent pigs are depicted in Table 4, whereby sensory attributes of LTL muscle in FE-divergent pigs are illustrated in Fig. 3. Intramuscular fat (IMF) content significantly differed between the FE groups (*P* < 0.05), with the high-FE carcasses having leaner muscle (1.49% IMF) comparing to low-FE carcasses (1.89% IMF). Muscle depth and percent lean meat did not differ significantly between the FE groups however pH at 45min *post-mortem* (*pm)* showed a tendency toward decreased values in the high-FE pigs (*P* < 0.1) while pH measured at 2h, 3h, 4h, 5h and 24h *pm* was significantly lower in the high-FE group (*P* < 0.05), and changes in pH evolution over time are depicted in Fig. 4. Drip loss did not vary between the FE groups. Muscle from high-FE pigs had increased cook loss on day 1 *pm* (*P* < 0.01) but there was no difference detected on day 7 *pm*. Although meat produced by high-FE pigs was significantly associated with increased Warner Bratzler shear force values (WBSF, less tender) on day 1 *pm* (*P* < 0.05) and had a tendency towards increased WBSF values on day 7 *pm* (*P* < 0.1), this difference in tenderness between the FE groups was not detected by sensory panellists. However, pork sensory assessment revealed that meat produced from high-FE pigs had higher scores for salty taste (*P* < 0.05) and a tendency towards increased barny/earthy/animal stable flavour (*P* < 0.1). Nutritive profile in relation to fatty acid (FA) proportions in LTL muscle (mg FA/100g meat), and percentage of FA in IMF of FE-divergent pigs are shown in Fig. 5. SFA did not differ significantly in LTL muscle of FE-divergent pigs, however, a tendency towards decreased proportions for each of palmitic and stearic acids in high-FE muscle was observed (*P* < 0.1). Muscle from high-FE group contained significantly lower amounts of the MUFA, palmitoleic acid (*P* < 0.05) and had a tendency towards decreased proportions of eicosenoic and oleic acids (*P* < 0.1). While PUFA content of muscle did not differ, when comparing the IMF *per se*, high-FE muscle had significantly greater concentrations of linoleic and alpha-linolenic acids (*P* < 0.05).

**Table 4:** Product quality traits of *Longissimus thoracis et lumborum* muscle divergent in feed efficiency (FE)

Trait	High-FE^a^	Low-FE^a^	SE	*P*-value
Fat depth (mm)	14.6	15.5	0.95	0.364
Muscle depth (mm)	54.6	56.8	2.36	0.367
Lean (%)	56.2	55.5	0.87	0.477
IMF (%)	1.49	1.89	0.19	0.046
Drip loss (%)	4.71	4.16	0.68	0.428
WBSF day 1 *pm* (N)^b^	37.0	31.8	2.33	0.036
WBSF day 7 *pm* (N)^b^	28.9	26.4	1.46	0.089
Cook loss day 1 *pm* (%)	36.4	34.0	0.67	0.001
Cook loss day 7 *pm* (%)	37.8	37.2	0.58	0.250

^a^Least square means for each trait; ^b^*WBSF* Warner Bratzler shear force (higher values indicate decreased tenderness).

**Fig. 6:**
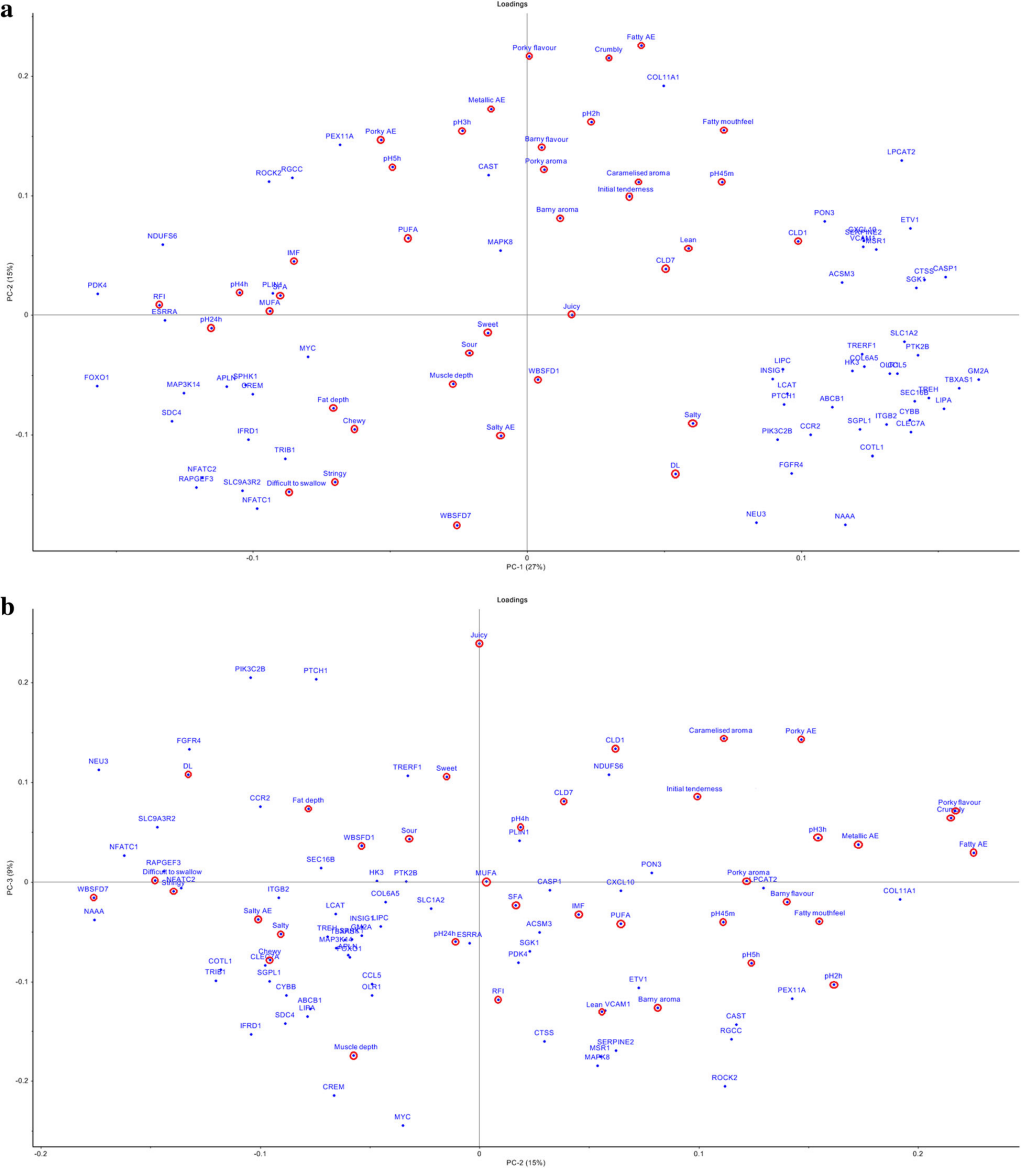
Principal component analysis of product quality traits with normalised expression values of selected DE genes, out of the 272, in FE-divergent pigs. PC1 vs PC2 is depicted in biplot (**a**) and PC2 vs PC3 is depicted in biplot (**b**). DL: drip loss (%), WBSF D1: Warner Bratzler shear force day 1 *pm* (N), WBSF D7: Warner Bratzler shear force day 7 *pm* (N), CL D1: cook loss day 1 *pm* (%), CL D7: cook loss day 7 *pm* (%), Fat depth (mm), Muscle depth (mm), Lean (%), IMF: intramuscular fat content (%), SFA: saturated fatty acid (mg), MUFA: monounsaturated fatty acid (mg), PUFA: polyunsaturated fatty acid (mg); AE: after effect; meat sensory attributes were scored from 0 (not detectable) to 100 (extremely detectable)

Furthermore, a number of significant correlations at a *P* < 0.05 were identified between product quality / sensory traits / nutritive value and genes differentially expressed in FE-divergent pigs (Tables 5 and 6). The strongest positive linear relationships were observed between fatty after effect and *COL11A1* (r = 0.76), percentage lean and *HK3* (r = 0.679), and cook loss on day 7 *pm* and *PON3* (r = 0.621). The strongest negative linear relationships were observed between cook loss on day 1 *pm* and *NFATC2* (r = -0.809), MUFA and *HK3* (r = -0.741), and cook loss on day 1 *pm* and *MYC* (r = -0.724). Principal component analysis (PCA) revealed that PC-1, PC-2 and PC-3 (Fig. 6a and b) cumulatively accounted for a total of 51 percent of the variation. PC-1, explaining 27 percent of the variation, was driven by our selected measure of FE, residual feed intake (RFI; the difference between actual feed intake and predicted feed requirements) and also captured the variability in certain meat quality traits, including ultimate pH, IMF and cook loss, which were associated with RFI (Fig. 2, Tables 4 and 5). PC-2, accounting for 15 percent of the variation, was not associated with RFI and captured some of the variation related to the product quality traits. As it can be seen in Fig. 6b, close co-locations were noted for WBSF day 7 *pm* and *NAAA,* pH 45m, 2h, 5h *pm* and *CAST*, pH 24h *pm* and *ESRRA*, pH 4h *pm* and *PLIN1*, cook loss day 1 *pm* and *NDUFS6*, drip loss and *FGFR4*, as well as lean percentage and *VCAM1*. Moreover, the biplot revealed a close co-location of a number of sensory attributes with genes i.e., porky aroma and L*PCAT2*, barny aroma and *ETV1*, sweet and *TRERF1*, difficult to swallow and *RAPGEF3*, stringy and *NFATC2* and *NFATC2*, as well as chewy and *CLEC7A*.

**Table 5:** Correlations between product quality traits and selected differentially expressed genes, out of the 272, in pigs divergent for feed efficiency.

Gene	pH 45m	pH 24h	DL	WBSF D1	WBSF D7	CL D1	CL D7	Fat depth	Muscle depth	Lean	IMF	SFA	MUFA	PUFA
*CCR2*	0	-0.51	0.44	0.01	0.16	0.2	0.06	0.01	-0.08	0.01	-0.38	-0.26	-0.22	-0.37
	0.99	**0.04**	0.09	0.98	0.56	0.45	0.83	0.96	0.77	0.97	0.15	0.34	0.41	0.16
*COL11A1*	0.44	-0.45	-0.13	0.04	-0.46	0.25	0.24	-0.32	-0.42	0.14	-0.03	0	-0.02	0.14
	0.09	0.08	0.64	0.88	0.07	0.36	0.38	0.23	0.11	0.6	0.92	1	0.93	0.59
*COL6A5*	0.34	-0.46	0.27	0.24	0.31	0.42	0.21	-0.11	0.12	0.29	-0.22	-0.41	-0.43	-0.18
	0.2	0.07	0.31	0.37	0.24	0.11	0.44	0.69	0.67	0.28	0.42	0.12	0.1	0.51
*CXCL10*	0.31	-0.25	0.22	-0.07	-0.13	0.37	-0.29	-0.34	0.04	0.36	-0.63	-0.61	-0.65	-0.46
	0.24	0.35	0.41	0.8	0.64	0.16	0.27	0.19	0.88	0.16	**0.01**	**0.01**	**0.01**	0.07
*CYBB*	0.28	-0.4	0.3	-0.04	0.21	0.11	-0.04	-0.44	0.06	0.5	-0.59	-0.53	-0.54	-0.36
	0.29	0.13	0.26	0.89	0.44	0.7	0.89	0.09	0.82	**0.04**	**0.02**	**0.04**	**0.03**	0.16
*FOXO1*	-0.43	0.52	-0.2	0.02	0.08	-0.63	-0.35	0.54	0.16	-0.53	0.26	0.5	0.62	0.09
	0.1	**0.04**	0.46	0.93	0.77	**0.01**	0.19	**0.03**	0.55	**0.04**	0.32	**0.04**	**0.01**	0.74
*GM2A*	0.39	-0.57	0.28	-0.14	-0.09	0.4	0.33	-0.41	-0.28	0.39	-0.33	-0.37	-0.43	-0.1
	0.14	**0.02**	0.29	0.61	0.73	0.12	0.22	0.12	0.29	0.14	0.22	0.16	0.1	0.71
*HK3*	0.09	-0.35	0.08	-0.23	-0.05	0.35	-0.31	-0.7	0.07	0.68	-0.54	-0.63	-0.74	-0.37
	0.75	0.19	0.76	0.4	0.85	0.19	0.24	**<0.01**	0.81	**<0.01**	**0.03**	**0.01**	**<0.01**	0.16
*LPCAT2*	0.35	-0.34	-0.11	-0.01	-0.25	0.48	0.29	-0.35	-0.16	0.27	-0.22	-0.20	-0.27	0.09
	0.18	0.20	0.68	0.98	0.35	0.06	0.27	0.18	0.55	0.32	0.41	0.46	0.31	0.73
*ETV1*	0.31	-0.37	0.09	0.30	0.11	0.38	0.23	-0.33	-0.04	0.33	-0.36	-0.34	-0.38	-0.13
	0.25	0.16	0.75	0.26	0.68	0.14	0.40	0.21	0.90	0.22	0.16	0.19	0.15	0.63
*NAAA*	-0.11	-0.33	0.47	-0.05	0.36	0.05	-0.09	-0.14	0.03	0.18	-0.44	-0.38	-0.38	-0.33
	0.69	0.22	0.07	0.86	0.18	0.86	0.74	0.61	0.93	0.51	0.08	0.15	0.14	0.21
*RAPGEF3*	-0.33	0.24	-0.08	0.18	0.47	-0.49	-0.06	0.40	0.12	-0.40	0.42	0.57	0.60	0.15
	0.21	0.36	0.76	0.50	0.07	0.05	0.83	0.13	0.66	0.12	0.10	**0.02**	**0.01**	0.59
*ITGB2*	0.36	-0.52	0.35	-0.09	0.1	0.41	0.06	-0.49	-0.14	0.53	-0.5	-0.54	-0.6	-0.37
	0.17	**0.04**	0.18	0.75	0.71	0.12	0.84	**0.04**	0.6	**0.04**	**0.04**	**0.03**	**0.01**	0.15
*LIPC*	-0.04	-0.08	0.24	0	0.27	0.15	-0.05	-0.06	0.41	0.17	-0.24	-0.36	-0.44	-0.28
	0.87	0.78	0.38	0.99	0.32	0.59	0.85	0.82	0.12	0.52	0.37	0.16	0.09	0.29
*MYC*	0.01	0.46	-0.21	-0.23	0.09	-0.72	-0.14	0.1	0.25	-0.03	0.24	0.28	0.26	0.2
	0.97	0.07	0.44	0.39	0.73	**<0.01**	0.6	0.7	0.36	0.92	0.38	0.29	0.32	0.47
*NEU3*	-0.24	-0.24	0.54	0.23	0.62	0.12	0.06	0.14	-0.11	-0.14	-0.06	-0.07	-0.06	-0.06
	0.38	0.38	**0.03**	0.39	**0.01**	0.65	0.84	0.62	0.69	0.61	0.84	0.8	0.81	0.82
*NFATC1*	-0.23	0.12	0.21	0.28	0.7	-0.49	-0.32	0.34	0.36	-0.24	0.14	0.21	0.25	-0.13
	0.39	0.65	0.42	0.29	**<0.01**	0.06	0.23	0.19	0.17	0.36	0.61	0.43	0.36	0.63
*NFATC2*	-0.29	0.38	0.07	-0.12	0.35	-0.81	-0.27	0.57	0.35	-0.44	0.28	0.38	0.46	-0.08
	0.28	0.15	0.8	0.66	0.18	**<0.01**	0.31	**0.02**	0.18	0.09	0.3	0.15	0.07	0.76
*PDK4*	-0.41	0.46	-0.59	0.14	-0.1	-0.27	-0.25	0.18	0.08	-0.25	0.33	0.48	0.51	0.26
	0.11	0.08	**0.02**	0.62	0.72	0.32	0.35	0.5	0.77	0.35	0.21	0.06	0.05	0.32
*PIK3C2B*	-0.02	-0.22	0.51	-0.09	0.16	0.13	0.27	0.22	-0.5	-0.4	0.06	0.19	0.21	0.18
	0.95	0.42	**0.04**	0.73	0.55	0.63	0.31	0.41	0.05	0.13	0.84	0.47	0.44	0.5
*PLIN1*	0.18	0.3	-0.49	-0.26	-0.32	-0.22	0.34	-0.02	-0.17	-0.11	0.51	0.61	0.61	0.59
	0.51	0.27	0.05	0.33	0.23	0.41	0.2	0.95	0.52	0.69	**0.04**	**0.01**	**0.01**	**0.02**
*PON3*	0.32	-0.3	-0.04	0.25	-0.14	0.56	0.62	-0.13	-0.31	0.07	-0.01	-0.06	-0.05	0.28
	0.23	0.26	0.88	0.34	0.62	**0.02**	**0.01**	0.63	0.24	0.8	0.98	0.82	0.86	0.3
*SDC4*	-0.32	0.54	-0.19	-0.04	0.21	-0.5	-0.58	0.14	0.41	0	-0.05	0.02	0.07	-0.22
	0.23	**0.03**	0.49	0.87	0.44	0.05	**0.02**	0.59	0.11	1	0.85	0.93	0.8	0.41
*SLC1A2*	0.09	-0.53	0.29	0.35	0.2	0.51	-0.24	-0.52	-0.23	0.44	-0.61	-0.47	-0.53	-0.29
	0.76	**0.04**	0.29	0.21	0.48	0.05	0.38	0.05	0.41	0.1	**0.02**	0.08	**0.04**	0.29
*TREH*	0.23	-0.27	0.45	-0.28	0.15	0.05	0.33	-0.06	-0.23	0.01	-0.16	-0.18	-0.19	0.02
	0.41	0.33	**0.09**	0.31	0.59	0.85	0.23	0.83	0.42	0.97	0.58	0.52	0.49	0.95

Correlation coefficient is presented in the upper row and a *P*-value is shown in the bottom row. Significant correlations are highlighted in bold. *DL* drip loss (%), *WBSF D1* Warner Bratzler shear force day 1 *pm* (N), *WBSF D7* Warner Bratzler shear force day 7 *pm* (N), *CL D1* cook loss day 1 *pm* (%), *CL D7* cook loss day 7 *pm* (%), Fat depth (mm), Muscle depth (mm), Lean (%), *IMF* intramuscular fat content (%), *SFA* saturated fatty acid (mg), *MUFA* monounsaturated fatty acid (mg), *PUFA* polyunsaturated fatty acid (mg)

**Table 6:** Correlations between product sensory traits and selected differentially expressed genes, out of the 272, in pigs divergent for feed efficiency

Gene/trait^a^	1	2	3	4	5	6	7	8	9	10	11	12	13	14	15	16	17	18	19
*CCR2*	-0.26	0.09	-0.30	-0.13	0.18	0.04	0.13	-0.23	-0.25	0.09	0.11	0.17	-0.37	-0.31	0.00	-0.24	0.23	-0.10	-0.14
	0.32	0.73	0.25	0.64	0.51	0.89	0.63	0.40	0.34	0.75	0.70	0.53	0.16	0.24	1.00	0.36	0.40	0.72	0.59
*COL11A*	0.16	0.33	0.07	-0.02	-0.25	-0.05	-0.24	0.52	0.35	-0.16	0.01	-0.14	0.48	0.25	0.00	0.18	-0.16	0.42	0.76
	0.56	0.21	0.81	0.95	0.34	0.86	0.36	0.04	0.18	0.55	0.97	0.62	0.06	0.34	0.99	0.51	0.54	0.10	**<0.01**
*COL6A5*	0.01	0.08	0.11	-0.12	0.20	0.10	-0.07	-0.07	0.15	0.19	0.10	0.27	-0.14	0.04	0.28	-0.13	-0.16	0.08	**-0.11**
	0.98	0.78	0.68	0.66	0.45	0.70	0.79	0.80	0.59	0.47	0.71	0.31	0.62	0.89	0.30	0.63	0.54	0.78	**0.70**
*CXCL10*	0.06	0.34	0.08	-0.11	-0.29	0.15	-0.02	0.29	0.50	0.09	-0.05	-0.03	0.23	0.10	-0.17	0.05	-0.56	0.40	0.39
	0.83	0.19	0.78	0.68	0.27	0.57	0.95	0.27	**0.04**	0.74	0.85	0.92	0.40	0.71	0.53	0.86	**0.02**	0.13	0.13
*CYBB*	-0.39	-0.16	0.15	-0.24	-0.21	0.04	-0.11	-0.28	0.10	0.02	0.36	0.00	-0.40	-0.15	-0.26	-0.59	0.14	-0.45	-0.14
	0.14	0.56	0.59	0.36	0.44	0.88	0.69	0.29	0.72	0.95	0.17	1.00	0.13	0.59	0.33	**0.02**	0.61	0.08	0.59
*FOXO1*	-0.23	-0.33	0.01	-0.11	-0.19	0.29	0.32	-0.36	-0.55	0.27	-0.18	0.13	-0.41	-0.18	0.18	0.05	0.36	-0.05	-0.38
	0.39	0.22	0.97	0.70	0.49	0.28	0.22	0.16	**0.03**	0.31	0.51	0.63	0.12	0.51	0.51	0.85	0.16	0.86	0.15
*GM2A*	-0.21	-0.11	-0.05	-0.06	0.02	-0.11	-0.17	-0.02	0.14	-0.09	0.51	0.16	-0.11	-0.15	-0.14	-0.43	0.15	-0.14	0.15
	0.43	0.69	0.85	0.81	0.93	0.69	0.53	0.93	0.62	0.75	**0.04**	0.56	0.67	0.57	0.59	0.09	0.57	0.60	0.57
*HK3*	-0.11	0.06	-0.17	0.03	-0.06	-0.21	-0.16	0.05	0.48	-0.31	0.30	-0.12	-0.09	0.05	-0.37	-0.34	-0.14	-0.11	0.05
	0.70	0.83	0.53	0.92	0.82	0.42	0.55	0.86	0.06	0.25	0.26	0.66	0.75	0.85	0.15	0.20	0.62	0.69	0.86
*LPCAT2*	0.31	0.52	0.07	0.09	-0.07	-0.17	-0.18	0.31	0.28	-0.34	-0.17	-0.30	0.33	0.21	-0.09	-0.02	-0.35	0.30	0.60
	0.24	**0.04**	0.79	0.74	0.80	0.53	0.50	0.24	0.29	0.19	0.53	0.25	0.22	0.43	0.75	0.94	0.18	0.26	0.01
*ETV1*	0.34	0.47	0.13	-0.20	-0.23	0.04	-0.10	0.21	0.37	-0.20	-0.15	-0.42	0.28	0.14	-0.05	-0.18	-0.47	0.10	0.46
	0.20	0.07	0.63	0.46	0.39	0.87	0.70	0.44	0.16	0.46	0.59	0.10	0.29	0.62	0.85	0.51	0.06	0.71	0.08
*NAAA*	-0.50	-0.35	-0.02	-0.10	0.01	0.02	0.11	-0.51	-0.11	0.04	0.34	-0.01	-0.65	-0.26	-0.13	-0.59	0.26	-0.43	-0.35
	**0.04**	0.18	0.94	0.70	0.98	0.93	0.68	**0.04**	0.68	0.89	0.20	0.97	**0.01**	0.33	0.63	**0.02**	0.32	0.10	0.18
*RAPGEF3*	-0.13	-0.20	-0.26	-0.43	-0.11	0.47	0.76	-0.65	-0.61	0.46	0.01	-0.12	-0.31	-0.38	0.34	-0.01	0.31	-0.15	-0.50
	0.63	0.46	0.33	0.09	0.67	0.07	**<0.01**	**0.01**	**0.01**	0.07	0.96	0.66	0.24	0.15	0.20	0.98	0.24	0.58	**0.04**
*ITGB2*	-0.26	-0.09	-0.15	-0.33	-0.12	0.15	0.10	-0.15	0.19	0.22	0.63	0.23	-0.12	-0.24	-0.16	-0.43	0.00	-0.14	-0.05
	0.32	0.73	0.57	0.21	0.66	0.58	0.70	0.57	0.47	0.41	**0.01**	0.39	0.66	0.37	0.54	0.09	0.99	0.62	0.87
*LIPC*	0.13	0.24	0.13	0.10	0.11	-0.10	-0.02	-0.04	0.25	-0.22	-0.40	-0.34	0.00	0.09	-0.14	0.06	-0.36	0.05	0.04
	0.64	0.36	0.63	0.72	0.69	0.71	0.95	0.90	0.36	0.41	**0.13**	0.20	1.00	0.75	0.61	0.82	0.17	0.85	0.89
*MYC*	-0.19	-0.48	0.20	-0.24	-0.42	0.18	0.09	-0.31	-0.21	0.10	0.21	-0.10	-0.26	-0.29	-0.02	-0.28	0.36	-0.43	-0.29
	0.49	0.06	0.45	0.37	0.11	0.51	0.73	0.24	0.44	0.72	0.43	0.72	0.32	0.28	0.93	0.29	0.18	0.10	0.28
*NEU3*	-0.35	-0.34	-0.15	0.06	0.46	0.04	0.29	-0.59	-0.23	0.25	0.20	-0.01	-0.56	-0.16	0.24	-0.25	0.18	-0.40	-0.59
	0.19	0.20	0.58	0.81	0.07	0.90	0.28	0.02	0.39	0.35	0.46	0.97	**0.03**	0.54	0.37	0.36	0.51	0.12	**0.02**
*NFATC1*	-0.29	-0.26	-0.03	-0.31	0.02	0.45	0.55	-0.68	-0.36	0.52	-0.17	-0.07	-0.52	-0.23	0.37	0.06	0.17	-0.23	-0.68
	0.27	0.33	0.90	0.25	0.95	0.08	**0.03**	**<0.01**	0.16	**0.04**	0.53	0.79	**0.04**	0.40	0.16	0.82	0.52	0.39	**<0.01**
*NFATC2*	-0.29	-0.25	-0.17	-0.26	0.00	0.27	0.37	-0.46	-0.66	0.43	-0.06	0.16	-0.46	-0.49	0.21	0.04	0.52	-0.41	-0.71
	0.27	0.35	0.53	0.34	0.99	0.32	0.15	0.07	**0.01**	0.10	0.81	0.56	0.07	0.06	0.42	0.90	**0.04**	0.12	**<0.01**
*PDK4*	0.11	-0.12	0.04	-0.16	-0.43	0.28	0.36	-0.17	-0.19	0.01	-0.14	-0.13	-0.03	0.13	0.12	0.03	0.00	0.34	0.07
	0.69	0.66	0.88	0.55	0.09	0.30	0.17	0.53	0.49	0.97	0.59	0.63	0.91	0.64	0.65	0.91	0.99	0.19	0.79
*PIK3C2B*	-0.14	0.11	-0.48	0.36	0.75	-0.26	0.14	-0.19	-0.43	0.13	0.05	-0.06	-0.19	-0.31	0.18	0.09	0.26	-0.41	-0.32
	0.62	0.68	0.06	0.18	**<0.01**	0.33	0.61	0.49	0.10	0.64	0.85	0.83	0.49	0.25	0.50	0.74	0.32	0.12	0.22
*PLIN1*	-0.12	-0.16	0.02	0.17	0.17	-0.18	-0.05	-0.18	-0.49	-0.04	0.09	0.14	0.01	-0.01	-0.09	0.12	0.54	-0.27	-0.13
	0.66	0.56	0.94	0.53	0.53	0.50	0.85	0.51	0.06	0.88	0.75	0.59	0.98	0.96	0.75	0.66	**0.03**	0.31	0.64
*PON3*	0.13	0.17	0.42	0.16	0.09	-0.14	-0.27	0.18	0.12	-0.13	-0.04	-0.01	0.24	0.39	0.03	-0.04	-0.21	0.19	0.45
	0.64	0.52	0.11	0.56	0.73	0.61	0.31	0.51	0.65	0.62	0.89	0.97	0.37	0.13	0.91	0.88	0.42	0.49	0.08
*SDC4*	-0.24	-0.45	0.10	-0.37	-0.48	0.41	0.34	-0.39	-0.16	0.34	0.17	0.19	-0.36	-0.19	-0.04	-0.24	0.11	-0.13	-0.47
	0.36	0.08	0.70	0.16	0.06	0.11	0.20	0.13	0.56	0.19	0.53	0.49	0.16	0.49	0.87	0.38	0.68	0.63	0.07
*SLC1A2*	-0.18	-0.12	-0.05	-0.14	-0.21	0.20	0.12	-0.20	0.44	0.00	0.27	-0.14	-0.25	0.13	0.01	-0.43	-0.33	0.17	0.16
	0.52	0.67	0.87	0.61	0.45	0.47	0.67	0.48	0.10	0.99	0.33	0.63	0.37	0.64	0.97	0.11	0.24	0.54	0.56
*TREH*	-0.29	-0.01	-0.04	-0.04	0.10	-0.10	0.08	-0.20	-0.21	0.14	0.33	-0.12	-0.20	-0.30	-0.04	-0.37	0.17	-0.46	-0.12
	0.30	0.97	0.87	0.87	0.72	0.73	0.77	0.48	0.46	0.61	0.24	0.68	0.47	0.27	0.88	0.18	0.55	0.09	0.66

Correlation coefficient is presented in the upper row and a *P*-value is shown in the bottom row. Significant correlations are highlighted in bold. ^a^1: porky aroma, 2: caramelised aroma, 3: barny aroma, 4: initial tenderness, 5: juicy, 6: chewy, 7: stringy, 8: crumbly, 9: fatty mouthfeel, 10: difficult to swallow, 11: salty, 12: sweet, 13: porky flavour, 14: barny flavour, 15: sour, 16: porky after effect, 17: salty after effect, 18: metallic after effect, 19: fatty after effect.

## Discussion

Functional annotation of divergent genes revealed a number of biological events related to immune response, growth, carbohydrate & lipid metabolism and connective tissue indicating that these might be important mechanisms governing differences in FE. Alongside attaining insights of the biological processes contributing to differences in muscle of FE-divergent pigs, we also investigated the consequences of the divergence in FE on product quality and the functional networks within muscle that underpin the relationship between FE and product quality. Divergence in FE affected various aspects of product quality and nutritive value, such as pH, tenderness, cook loss, as well as IMF content and fatty acid proportions, and this study provided clues with regards to biological mechanisms driving the relationship between FE and the observed alteration in product quality traits of economic significance.

### Immune response

Ontology analysis revealed a number of pathways and biological functions related to immune response as being relevant to FE in porcine muscle. Greater amounts of leucocytes [21] and higher cellular immune response [22], were previously observed in pigs selected for lean growth. In the present study ‘protein kinase C-theta (PKCθ) signalling in T lymphocytes’ and ‘cluster of differentiation 28 (CD28) signalling in T helper cells’, which activate and promote differentiation of T cells, were significantly enriched cell-mediated immune responses. These features were also observed in pigs selected for lean growth that were subjected to an immunological challenge i.e. tetanus toxoid [22]. Furthermore, ‘tec-kinase signalling’, involved in development and function of cellular immune response T-cells [23] was significantly activated (z-score = 2.12) in high-FE pigs exhibiting leaner growth. This pathway, alongside many other significantly over-represented pathways, was enriched with Phosphatidylinositol-4-Phosphate 3-Kinase Catalytic Subunit Type 2 Beta (*PIK3C2B*) belonging to a family of enzymes modulating immune cell development, differentiation and function [24]. *PIK3C2B* was specifically shown to play a key role in T-cell activation [25]. Functional annotation also exposed ‘Interleukin 8 (IL-8) signalling’, controlling trafficking of neutrophils and macrophages to the site of inflammation [26], to be significantly activated in high-FE pigs (z-score = 2.50). ‘Accumulation of phagocytes’ and ‘phagocytosis’, contained within the broader ‘immune cell trafficking and tissue development’ and ‘cellular function and maintenance’ categories, respectively, were also predicted to be significantly activated in high-FE pigs (z-score = 2.15 and 2.12, respectively). Additionally pathways playing a role in stimulating phagocytes activity [27] and muscle repair capabilities [28], ‘N-formyl-Met-Leu-Phe (fMLP) signalling in neutrophils’ and ‘production of nitric oxide in macrophages’, tended towards activation in high-FE pigs. Fuelling immune response is an energetically expensive process, which would be suspected to lower animal’s feed efficiency due to prioritizing nutrients towards the immune-related processes [8]. Previous literature has reported decreased immune response in muscle from high-FE pigs [18]. On the contrary, a study conducted in cattle identified a number of immune-related processes, representing both innate and adaptive response, significantly activated in muscle of high-FE individuals i.e., ‘immune response of antigen presenting cells and leukocytes’, ‘response of mononuclear leukocytes and myeloid cells’, and ‘immune response of phagocytes’ [29]. Here, our findings suggest that muscle from high-FE pigs exhibit activated immune response. Furthermore, more reliance on adaptive rather than innate immunity, which could reduce feed requirements, may stimulate faster growth of muscle [30] from high-FE pigs.

### Carbohydrate metabolism and glycolytic potential

pH evolution in the *pre-rigor* period was highly divergent in relation to FE status. At the earliest time-point measured (45m *pm*), pH did not significantly differ. However, pH at all later stages in the early *pm* period- up to 5 hours- as well as ultimate pH monitored the next day significantly differed between the two groups, with the high-FE pigs showing decreased pH values in the muscle. This is consistent with previous studies demonstrating greater glycolytic potential in high-FE pigs, wherein pH 30m *pm* did not differ but ultimate pH was significantly reduced in high-FE pigs [13, 16]. Low pH in meat can increase the perception of sour taste due to a higher concentration of free hydrogen ions [31, 32]. Nevertheless, the significant difference in ultimate pH was not detected as increased acidity by sensory panellists in this study, who perceived no difference in sour taste of meat from high- versus low-FE. However, the reduced ultimate pH may have contributed to significantly higher scores for ‘salty taste’ that were observed in high-FE meat. This is in agreement with Lipinski et al. [33] who previously found that meat with lower pH can be perceived by sensory panellists as more salty.

Genes involved in glycolysis and energy metabolism were previously reported to be up-regulated in chickens exhibiting lower ultimate pH [34]. Here, pH evolution was significantly different in the FE-divergent muscle, and trehalase (*TREH*) that codes for an enzyme catalysing the conversion of trehalose to glucose [35] was the most up-regulated gene (fold change = 4.49) in high-FE pigs. This might indicate that this group of pigs could potentially exhibit more efficient energy conversion in growth, but with potential consequences for *post-mortem* energy metabolism and product quality. In support to this, PCA revealed negative correlations between *TREH* and pH at 45m up to 5h *pm*, whilst a positive correlation between *TREH* and the discussed above product saltiness (Fig. 6b). Indeed, ontology analysis highlighted the molecular function ‘catabolism of oligosaccharides’, which was enclosed within a broader ‘carbohydrate metabolism’ category, as being highly relevant to the gene expression changes in divergent FE muscle. Moreover genes enriched in this sub-category, *GM2* ganglioside activator (*GM2A*, fold change = 1.60; high-FE > low-FE) and neuraminidase 3 (*NEU3*, fold change = 1.29; high-FE > low-FE), were negatively correlated with ultimate pH and positively correlated with drip loss, respectively (Table 5). These findings suggest that differences in carbohydrate conversion into glucose underpin the differential evolution of pH profile in FE-divergent muscle.

Carbohydrate metabolism has an important influence on water-holding capacity of meat [36]. Water-holding capacity traits, alongside tenderness, are closely linked with pH and here unfavourable associations between FE, lower ultimate pH and increased cook loss on day 1 *pm* with a reduced tenderness (6N increase in WBSF) were observed. However, sensory tenderness remained unchanged and, other than saltiness, no other sensory attributes were altered. It has been previously postulated that lower water-holding capacity of meat with decreased ultimate pH can result in tougher beef (increased WBSF) [37], which is consistent with our (WBSF) observations in the present study. Another gene that was associated with tenderness and aspects of pH profile and water-holding capacity was calpastatin (*CAST),* the inhibitor of calpain*. CAST* was closely located to pH 45m, 2h and 5h *pm* (Fig. 6b), and this is consistent with its known associations with water-holding capacity in pork [38]. Another potential candidate gene for quality is transcription factor P64 (*MYC*), which plays a role in apoptosis [39]. In the present study, a correlation analysis (Table 5) demonstrated a negative correlation between *MYC* and cook loss on day 1 *pm* and this is consistent with a previous study which identified a SNP in *MYC* to be associated with pH and cook loss in pork [40].

### Growth

Syndecan-4 (*SDC4)* was the most down-regulated gene (fold change = -2.35) in high-FE muscle. *SDC4* is a gene encoding plasma membrane proteoglycans and has been previously shown to have an impact on muscle cell proliferation and differentiation [41]. Knock down of *SDC4* has been associated with increased myogenic regulatory transcription factor [42] and myogenin expressions, as well as increased muscle differentiation [43], which signify its importance to muscle growth. Integrating functional annotations of DE genes revealed a number of biological processes related to growth. ‘Tissue development’ and ‘cardiovascular system development & function’ were significantly enriched categories amongst the DE genes, with forkhead box O1 (*FOXO1*) being included (fold change = -1.49) in both categories. FOXO1 belongs to the FOXO forkhead type family of transcription factors and it plays a role in modulation of skeletal muscle angiogenesis and function [44]. Mice over-expressing *FOXO1* were found to weigh less and had a decreased skeletal muscle mass [45].

‘Mobilisation and flux of Ca^2+’^, contained within a ‘vitamin and mineral metabolism’ category, were significantly activated in high-FE pigs (z-score = 2.9 and 2.4, respectively). Calcium plays a key role in function and plasticity of skeletal muscle. It regulates skeletal muscle formation [46, 47], homeostasis and regeneration as well as being a crucial component triggering muscle contraction that enables movement [47] and furthermore plays an important role *post-mortem* in tenderness development [48]. Moreover ‘synthesis of alpha-amino acids’ and ‘catabolism of L-tryptophan’, enclosed within an ‘amino acid metabolism’ category, as well as ‘production of protein’, contained within ‘protein metabolism’ category, were significantly enriched amongst DE genes. L-tryptophan is an alpha-amino acid that positively influences production of protein in skeletal muscle and growth performance [49]. L-tryptophan is also a precursor of a broad range of compounds regulating appetite therefore playing a role in FE [49]. Although muscle and adipose depth did not significantly differ between the FE groups, down-regulation of *SDC4* & *FOXO1,* altered amino acid metabolism and increased mobilisation & flux of Ca2+ may impact, at least to some extent, growth in FE-divergent pigs.

### FE, connective tissue and tenderness

Collagen type XI alpha 1 chain *(COL11A1)* and collagen type VI alpha 5 chain (*COL6A5)* were up-regulated (fold change = 1.52 and 1.77, respectively) in high-FE pigs. Over-expression of *COL11A1* has been associated with decreased tenderness in heifers [50]. Also, a single-nucleotide polymorphism in this gene was identified to have a consistent association with meat tenderness across three cattle breeds [51]. This study highlights that its relevance to tenderness development is also conserved in porcine muscle. Ontology analysis highlighted several functions also related to connective tissue, for example ‘apoptosis of fibroblast cell lines’, enclosed within ‘cell death and survival’ category, was significantly inhibited in high-FE pigs (z-score = -2.27). All of the DE genes represented in this category were also enriched in ‘cell death of connective tissue’ (z-score = -1.18), which also falls under the broader ‘cell death and survival’ function. Apoptosis and the stress response have been implicated as important factors in tenderisation. Specifically, apoptosis and cell death is considered the first step in promotion of tenderisation and factors which down-regulate apoptosis, such as heat shock protein expression can inhibit tenderisation [52–54]. In the present study, the more efficient pigs produced muscle that tenderised more slowly, with significantly tougher pork on day 1 *pm* compared with less efficient counterparts. Even by day 7 *pm*, while the differences in shear force were small, a tendency towards increased toughness remained. In this scenario the modulation of apoptosis as observed through our gene expression studies may have contributed to this differential ageing associated with FE and should be a matter of consideration in further driving improvements in FE. Fibroblasts are the key players in the synthesis of extracellular matrix components such as collagen [55, 56]. Amongst down-regulated transcripts in muscle of high-FE pigs were nuclear factor of activated T-cells 1 (*NFATC1*), nuclear factor of activated T-cells 2 (*NFATC2*) and *MYC* (fold change = -1.30, -1.25 and -1.57, respectively) that have previously been shown to induce apoptosis in fibroblasts [39, 57, 58]. Correlation analysis between DE genes and product quality traits showed a negative correlation between *NFATC2* and cook loss on day 1 *pm*, whilst *NTAFC1* had a tendency towards being negatively correlated with cook loss on day 1 *pm*. Consistent with a role for apoptosis in tenderness development [54], *NFATC1* was also positively correlated with a measure of toughness, WBSF on day 7 *pm* (higher values indicate decreased tenderness). PC analysis revealed a positive correlation between *NFATC2* and stringiness (Fig. 6b). Moreover, WBSF was correlated with N-acylethanolamine acid amidase (*NAAA)*, a pro-inflammatory gene closely related to acid ceramidase [59], which has been shown to influence locomotory behaviour [60]. This may explain the positive correlation between *NAAA* and texture, known to be associated with load-bearing and functionality of specific muscles, with more load-bearing and active muscle producing tougher meat [61]. PC analysis further highlighted several other genes in relation to meat quality. Specifically, rap guanine nucleotide exchange factor 3 (*RAPGEF3*; involved in angiogenesis [62]) and *NFATC1* co-located with texture traits, including WBSF, stringiness and difficulty in swallowing, whilst crumbliness was situated on the opposite end of the biplot (Fig. 6b). These observations were also noted in the correlation analysis (Table 6). With a complex array of correlated traits, and numerous differentially expressed genes in consideration, this study illustrates the utility of a holistic multivariate approach such as PCA in identifying novel candidates for association with traits of interest.

Besides connective tissue, tenderness of meat has been shown to be influenced by greater calpastatin activity through decreased *pm* protein degradation [17], In the present study, calpastatin (*CAST*) had a tendency towards being down-regulated (*P* < 0.1, fold change = -1.17) in high-FE pigs which is not expected given our observation that tenderness was impaired in high-FE pigs, furthermore tenderness development was slower in this group. This negative correlation between *CAST* and tenderness was also demonstrated in the PCA biplot with WBSF (day 1 and 7 *pm*) and *CAST* being located on opposite ends of PC-2. While calpastatin has been shown to be negatively associated with tenderness, most of this work has been done in beef [63–66] and its relationship with pork quality may be more important in relation to water-holding capacity [38]. Nevertheless, the altered tenderness of FE-divergent meat could be partially impacted by shifts in collagen turnover resulting from decreased degradation of fibroblasts.

### Lipid metabolism changes associated with FE

Muscle depth and leanness did not differ between the FE groups, which contrast previous reports [13, 14, 16, 17]. However, here and in prior studies, selection for high-FE was associated with reduced IMF [14, 16, 17]. Over the past decades, consumers have become more conscious with regards to wholesome eating and seeking healthier options [6]. Meat from high-FE pigs exhibited tendencies towards decreased levels of SFA and MUFA, which are known to be the major constituents of triacylglycerol [67, 68] and are associated with increased risk of cardiovascular disease [69, 70]. Higher proportions of PUFA, whilst lower levels of SFA and MUFA, has been previously associated with reduced IMF content [68, 71, 72]. Indeed, the IMF of meat from high-FE pigs was 12 percent richer in PUFA compared to IMF from low-FE group. PUFA has been shown to reduce low-density lipoprotein cholesterol levels and exhibit protective properties against cardiovascular disease [15], therefore suggesting that meat from high-FE pigs may have a healthier fatty acid profile. Underpinning these changes, functions and pathways important in metabolism of lipids were also affected by FE, as evident from the ontology analysis, specifically ‘concentration of lipids, cholesterol & triacylglycerol’ and ‘fatty acid metabolism’, enclosed within a broader ‘lipid metabolism’ category. Correlation analysis between DE genes, enriched in ‘lipid metabolism’ category, and product quality traits revealed a number of significant correlations. *FOXO1* (fold change = -1.49), which was previously shown to play a role in adipogenesis in cattle [73], was positively correlated with fat depth, SFA and MUFA, and also negatively correlated with lean percentage. Cytochrome B-245 beta chain (*CYBB;* fold change = 1.56) was positively correlated with percent lean and negatively correlated with IMF, SFA and MUFA. A previous study conducting expression profiling of porcine adipose tissue suggested CYBB to play a role in fat metabolism and adipogenic differentiation [74]. Correlation analysis has also revealed Perilipin 1 (*PLIN 1*; fold change = -1.42) to be positively correlated with IMF, SFA, MUFA and PUFA and this is in keeping with a previous study reporting its higher abundance being associated with increased IMF in porcine muscle [75]. Moreover, C-X-C motif chemokine ligand 10 (*CXCL10*, fold change = 2.24), which was previously associated to marbling in cattle [76], was negatively correlated with IMF, SFA and MUFA.

Furthermore, the second most significant network (network #2), identified through the functional annotation analysis, contained several features related to ‘lipid metabolism’, ‘molecular transport‘ and ‘protein synthesis’. Paraoxonase 3 (*PON3*) and triacylglycerol lipase (*LIPC*) were the most up-regulated genes in this network (fold change = 4.40 and 3.51, respectively). PON3, an enzyme belonging to the PON family, associates with high density lipoproteins (HDL) [77], which are lipid particles that function to export excess cholesterol from muscle and adipose tissue to the liver [78]. PON3 knockout mice have previously been shown to exhibit increased body weight [79], which points towards a PON3 role in promoting a leaner muscle growth. The enzyme LIP catalyses hydrolysis of phospholipids and triacylglycerols [80]. Over-expression of *LIPC* in high-FE muscle suggests enhanced lipid degradation in this group of pigs and potentially underlies a more efficient fat metabolism in these animals.

## Conclusions

Gene expression profiling of muscle from FE-divergent pigs provided mechanistic insights on the biological events prevailing differences in FE, which impact product quality. Small but significant changes in the quality of meat, in relation to texture and water-holding capacity, from high-FE pigs, were observed. High-FE muscle was characterised by reduced intramuscular fat content and improved nutritional profile in terms of fatty acid composition. Ontology analysis revealed enhanced activity of adaptive immunity and phagocytes in high-FE pigs, which may indicate that these animals are more efficient in conserving resources that can be utilised for other important biological processes. Shifts in carbohydrate conversion into glucose in FE-divergent muscle may underpin the altered evolution of pH profile in meat from the divergent groups. Although muscle depth did not significantly differ between the FE groups, our transcriptomic findings indicate that altered amino acid metabolism and increased mobilisation & flux of calcium may influence, at least to some extent, growth in FE-divergent muscle. Moreover, decreased degradation of fibroblasts, the key players in the synthesis of the extracellular matrix, could impact on collagen turnover and alter tenderness of meat. Biological functions important in metabolism of lipids were also affected by FE. Specifically, enhanced lipid degradation in more efficient pigs may potentially underlie a more efficient fat metabolism in these animals.

## Materials and Methods

### Animals and experimental design

Animal housing, diets and tests were previously described in details in Metzler-Zebeli and colleagues [81]. 138 pigs from the intact litters of 12 sows (Landrace x Large White; Hermitage Genetics, Kilkenny, Ireland) inseminated with semen from 6 boars (Maxgro; Hermitage Genetics; 2 litters per boar, each having a high estimated breeding value for FE), were utilised in this study. Pigs, weaned at 28 days of age and group-housed (entire sibling groups), were provided with *ad libitum* access to feed and water. Diets were provided in the same sequence with the same ingredient and chemical composition (starter, link, weaner and finisher) and were delivered to pigs via Feed Intake Recording Equipment (FIRE) feeders (Schauer Agrotonic, Wels, Austria). Pigs were tested from day 42 until 91 post-weaning. Feed intake was recorded daily, whereas pig weight, back-fat depth and muscle depth were recorded weekly between day 70 and day 120 of age. Average daily feed intake (ADFI) and average daily gain (ADG) were calculated for each pig weekly. Residual feed intake (RFI, a measure of FE defined as the difference between actual feed intake and predicted feed requirements) was calculated after day 120 of age as the residual from a least squares regression model of ADFI on ADG, metabolic live weight, gender and also all relevant two-way interactions, and the effects of back-fat and muscle depth using the PROC REG procedure in SAS (version 9.4; SAS Inst. Inc., Cary, NC, USA). Based on RFI values, pigs were categorised within litter and gender as low (L) RFI and high (H) RFI and of these a total of 40 (20 extremes from LRFI (high-FE) - 10 males and 10 females, and 20 extremes from HRFI (low-FE) - 10 males and 10 females) were selected for gene expression profiling and meat quality analysis. The mean RFI (g/day) of the LRFI and HRFI pigs was -100.2 (SD: 97.9) and 150.7 (SD: 163.3) respectively, whereas the mean of feed conversion ratio (FCR, ratio of feed intake and weigh gain) of the LRFI (high-FE) and HRFI (low-FE) pigs was 1.98 (SD: 0.16) and 2.27 (SD: 0.25) respectively. The slaughter of animals, fasted for 18 hours with an average final body weight of 99 kg (SD: 11.4kg), occurred on 2 slaughter days, a week apart, and was by electronic stunning followed by exsanguination. Samples of the LTL muscle were collected and snap frozen in liquid nitrogen within 10 minutes *pm* followed by storage at -80°C until RNA isolation. The LTL muscle was excised 24 hours *pm* from each carcass and utilised for meat quality analysis.

### RNA library preparation, differential expression analysis and functional annotation

Sixteen muscle samples selected from the most FE-divergent siblings of the same gender (8 from LRFI (high-FE) - 4 males and 4 females, and 8 from HRFI (low-FE) - 4 males and 4 females), were snap frozen following which they were ground into fine powder in liquid nitrogen. Total RNA was isolated using Tri-Reagent (Sigma-Alrich, Taufkirchen, Germany), followed by DNase treatment and a column-based purification using the Nucleospin RNA II kit (Macherey-Nagel, Düren, Germany). RNA library preparation was carried out using the TruSeq Stranded mRNA protocol. Following RNA sequencing with Illumina HiSeq2500, paired-end reads were mapped to the reference Sscrofa10.2 (Ensembl release 84) [82] using TopHat (2.1.0). Read counts were assigned to the gene features using the HTSeq 0.6.1 program [83]. Differential gene expression analysis in relation to FE was performed using DESeq2 package (3.4.0, https://www.r-project.org), including RFI groups and sow as fixed effects. Gene symbols for significantly altered genes (*P* < 0.01) and related fold changes were submitted to Ingenuity Pathway Analysis (IPA; Ingenuity® Systems, http://www.ingenuity.com), whereby Benjamini-Hochberg (B-H) corrected *P*-values were used to detect significantly enriched bio-functions and canonical pathways (*P* < 0.01). Functional annotations with a z-score greater than 2 and lower than -2 were considered significantly activated and inhibited in high-FE pigs, respectively. Information enclosed in the Ingenuity® Knowledge Base was utilised to generate potential important interaction networks amongst the DE genes.

### Validation of RNA sequencing results

For cDNA synthesis, 1 μg of total RNA was utilized in the presence of random primers (Promega, Mannheim, Germany), oligo (dT) primer and Superscript® III reverse transcriptase (Invitrogen Corp., San Diego, CA, USA). Thirteen DE genes were selected for validation through quantitative real-time PCR (qPCR). Primers for target genes (Additional file 4: Table S4) were designed using Primer-BLAST software in the NCBI (https://www.ncbi.nlm.nih.gov/tools/primer-blast) based on *Sus scrofa* nucleotide sequences and their specificity was determined with the BLAST search tool database (http://www.ncbi.nlm.nih.gov/BLAST). qPCR was carried out with LightCycler 96 system (Roche Mannheim, Germany). 2 μl of cDNA was amplified in a 10 μl reaction volume using 6 μl SYBR Green I Master (Roche) and 0.6 μl (10 μM) of each forward and reverse primer. Cycling conditions for reference and DE genes consisted of initial denaturation at 95 °C for 5 min and 45 cycles of amplification (95 °C for 10 sec, 60 °C for 15 sec and 72 °C for 25 sec). A melting curve analysis was included at the end of the amplification to confirm the specificity of all amplification reactions. Normalised qPCR data were analysed using ANOVA test in R, including RFI groups as a fixed effect and sow as a random effect. Correlation analysis between the RNA-seq and qPCR data were carried out with R package considering the results as significant at *P* < 0.05.

### Product quality

Carcass grading along with technological and sensory meat quality traits as well as nutritional profiling of meat were measured using methods as described in detail by Horodyska and colleagues [14]. The carcass grading included fat depth, muscle depth, lean percent and IMF content, whilst the technological meat quality included pH (45m, 2h, 3h, 4h, 5h and 24h *pm*), drip loss, cook loss and tenderness (WBSF). Fatty acids were profiled to assess the nutritive value of meat. PROC MIXED procedure in the SAS system was used to evaluate associations between FE and meat quality traits in the Maxgro x (Landrace x Large White) pigs (*n* = 40). The model included RFI groups, gender & slaughter day as fixed effects, sow as a random effect, live weight as a covariate and the absolute values of RFI as a weight statement. Moreover, Spearman’s correlations (r) as well as principal component analysis of product quality / sensory traits / nutritive value and normalised expression values of selected DE genes, out of a total number of identified DE genes in RFI-divergent pigs (*n* =16), were determined using the PROC CORR procedure in the SAS system (version 9.4) and Unscrambler® software (version 10.3; CAMO software, Oslo, Norway), respectively.

## Additional files

Additional file 1: **Table S1.** Differentially expressed transcripts (*n* = 272) at a *P* < 0.01 between high-FE and low-FE groups. (XLSX 29 kb)

Additional file 2: **Table S2.** Biological functions significantly enriched with differentially expressed genes, including a list of sub-categories contained within each function. (XLSX 18 kb)

Additional file 3: **Table S3.** All canonical pathways significantly enriched with differentially expressed genes. (XLSX 12 kb)

Additional file 4: **Table S4.** Forward and reverse primers for RNA-seq validation through qPCR. (DOCX 16 kb)

## Abbreviations

FE: Feed efficiency
RFI: Residual feed intake
LTL: *Longissimus thoracis et lumborum*
IMF: Intramuscular fat content; *pm: post-mortem*
LRFI: Low RFI
HRFI: High RFI
WBSF: Warner Bratzler shear force
SFA: Saturated fatty acids
MUFA: Monounsaturated fatty acids
PUFA: Polyunsaturated fatty acids
