# Supplementary material for: RNA-seq of muscle from pigs divergent in feed efficiency and product quality identifies differences in immune response, growth, and macronutrient and connective tissue metabolism
Source: BMC Genomics. 2018 Nov 1;19:791. doi: 10.1186/s12864-018-5175-y (PMC6211475; doi:10.1186/s12864-018-5175-y)
Supplement: Supplementary file 4 — Table S4. Forward and reverse primers for RNA-seq validation through qPCR. (DOCX 16 kb) [file 12864_2018_5175_MOESM4_ESM.docx]

**Table S1** Forward and reverse primers for RNA-seq validation through qPCR.

| Gene | NCBI accession no. | Forward | Reverse | Product size |
| --- | --- | --- | --- | --- |
| *TRIM63* | NM_001184756.1 | AAAGTGTGCCAACGACATCTTC | GATGTTCTCCACCAGCAGATTC | 169 bp |
| *FOXO1* | NM_214014.2 | AGCTTCCCACACAGTGTCAAG | AGACATCTTTGGACTGCTTCTCTC | 134 bp |
| *FAM134B* | XM_005672396.2 | ACGGGACCTTCAACCTTTCAG | TTGTTCCCATGCCATTTTCTAG | 132 bp |
| *SPP1** | NM_214023.1 | TTGCTAAAGCCTGACCCATCT | CGTCGTCCACATCGTCTGTT | 145 bp |
| *CASP1* | NM_214162.1 | GCCATTAAGAAAGCCCACATAG | ACAGGACCAGGCATGTTCTTG | 144 bp |
| *COL11A1* | XM_001929372.7 | CTCCAGGTCAGCAAGGAAATC | GCCTTCTTTTCCAGGGTGAC | 152 bp |
| *CYBB* | NM_214043.2 | CTTGTACGGACGGCCTAACTG | CTTGTACGGACGGCCTAACTG | 144 bp |
| *ITGB2* | NM_213908.1 | ACGTGTGCCAGTGTGACTTTG | AGCTTAGTGGTCCCGCACTC | 160 bp |
| *MYC* | NM_001005154.1 | CACGACTCGCTCCTCTGAAAG | GTTGGTGAAGCTGACGTTGAG | 111 bp |
| *PLCB2* | XM_021097762.1 | GACGCAGCCACTCAACCATTAC | TTTCCCTTCCAACAGTCCAGCT | 150 bp |
| *SDC4* | XM_021077291.1 | TGGTCTGGCTCTGGAGATCTG | TCCTCCAGTTCCTTGGGTTC | 146 bp |
| *TREH* | XM_021062945.1 | TGATCTCAATGCCTTCCTGTG | GTCAAACCAGGCACCTTTCTC | 163 bp |
| *VCAM1* | NM_213891.1 | CATAAAGTCCAAGGAAACGAAGAG | ATAACTGGGTCCTTGGGTGAG | 168 bp |
| *RPL10* | NM_001044543.1 | CTGTGTTCGTCTTTTCTTCC | TCATCCACTTTTGCCTTCT | 199 bp |
| *RPL32* | NM_001001636.1 | AGCCCAAGATCGTCAAAAAG | TGTTGCTCCCATAACCAATG | 165 bp |

* Muráni, E., Ponsuksili, S., Seyfert, H.-M., Shi, X., & Wimmers, K. (2009). Dual effect of a single nucleotide polymorphism in the first intron of the porcine *Secreted phosphoprotein 1* gene: allele-specific binding of C/EBP beta and activation of aberrant splicing. *BMC Molecular Biology*, *10*, 96.
